# TDP-43–immunity–microbiota axis in amyotrophic lateral sclerosis: A potential pathogenic mechanism

**DOI:** 10.4103/NRR.NRR-D-25-00440

**Published:** 2025-09-03

**Authors:** Yasmine Abbassi, Dorian Fink, Francesco Cei, Elena Niccolai, Amedeo Amedei

**Affiliations:** 1 Department of Experimental and Clinical Medicine, University of Florence, Florence, Italy; 2 Laboratorio Congiunto MIA-LAB (Microbiome-Immunity Axis Research for a Circular Health), University of Florence, Florence, Italy; 3 Network of Immunity in Infection, Malignancy and Autoimmunity (NIIMA), Universal Scientific Education and Research Network (USERN), Florence, Italy

**Keywords:** amyotrophic lateral sclerosis, immunity, microbiome, neuroinflammation, short-chain fatty acids, TAR DNA-binding protein 43

## Abstract

Amyotrophic lateral sclerosis is a devastating neurodegenerative disease marked by progressive motor neuron degeneration. Despite extensive research, effective treatments remain elusive, underscoring the need to explore the molecular mechanisms driving disease progression. The amyotrophic lateral sclerosis complexity is further compounded by its large heterogeneity, encompassing both genetic and sporadic forms, diverse phenotypic presentations, and highly variable progression rates. A key pathological feature of amyotrophic lateral sclerosis is the aggregation of TAR DNA-binding protein 43, which contributes to cellular toxicity, neuroinflammation, and neuronal dysfunction. This review explores the complex interplay between TAR DNA-binding protein 43 pathology, immunity dysregulation, and the gut-brain axis, with a focus on the role of microbiome-derived metabolites in amyotrophic lateral sclerosis. Neuroinflammation, mediated by both innate and adaptive immunity, plays a central role in disease pathogenesis, with TAR DNA-binding protein 43 influencing immune signaling and exacerbating neurotoxicity. Additionally, disruptions in gut microbiota composition and intestinal barrier integrity, frequently observed in amyotrophic lateral sclerosis patients, suggest a potential role for the gut-brain axis in modulating neurodegenerative processes. By integrating evidence from emerging studies, our aim is to clarify how TAR DNA-binding protein 43 aggregation contributes to neuroinflammation and immune dysfunction while exploring the gut microbiota role as both a modulator and potential biomarker of disease. Understanding these interactions could pave the way for novel therapeutic strategies, including microbiome-targeted interventions such as probiotics, dietary modifications, or immune-modulating therapies. Finally, unraveling the TAR DNA-binding protein 43–immune system–microbiome axis may offer new avenues for personalized treatments aimed at mitigating neuroinflammation, slowing amyotrophic lateral sclerosis progression, and improving patient outcomes and life quality.

## Introduction

Amyotrophic lateral sclerosis (ALS) is a progressive neurodegenerative disease characterized by the selective loss of upper and lower motor neurons (MNs), leading to paralysis and death typically within 3–5 years of symptom onset, often due to respiratory failure (Rowland and Shneider, 2001; Hardiman et al., 2011). Approximately 10% of cases are familial ALS, commonly linked to mutations in genes such as *SOD1*, *FUS*, *C9orf72*, and *TARDBP* (Valdmanis and Rouleau, 2008; Taylor et al., 2016), while the remaining cases are sporadic ALS, where mislocalization and cytoplasmic aggregation of Transactive Response (TAR) DNA-Binding Protein 43 (TDP-43) are pathological hallmarks observed in 97% of patients (Neumann et al., 2006; Strong, 2010; Ling et al., 2013).

TDP-43, an RNA-binding protein, is essential for RNA processing and axonal mRNA transport (Alami et al., 2014). In ALS, its mislocalization from the nucleus to the cytoplasm leads to both loss of function and toxic gain-of-function effects, contributing to neurodegeneration (Taylor et al., 2016, Zhao et al., 2018). These pathological changes are often accompanied by neuroinflammation (NI), driven by activated glial cells and peripheral immune cell infiltration (McCombe et al., 2020; Niccolai et al., 2024b), as well as dysregulated adaptive immune responses involving T helper cells and regulatory T cells (Giovannelli et al., 2020; Jin et al., 2020). Emerging evidence also implicates the gut microbiome (GM) in ALS pathophysiology. Studies in animal models and humans reveal gut dysbiosis, barrier dysfunction, and decreased short-chain fatty acid–producing bacteria, all of which modulate systemic and central immune responses (Figueroa-Romero et al., 2019; Burberry et al., 2020; Niccolai et al., 2021, 2024; Mazzini et al., 2021). Microbiota interventions in ALS models, including butyrate supplementation and fecal microbiota transplantation, have shown potential in attenuating inflammation and disease progression (Zhang et al., 2017; Figueroa-Romero et al., 2019; Burberry et al., 2020).

**Figure 1:**
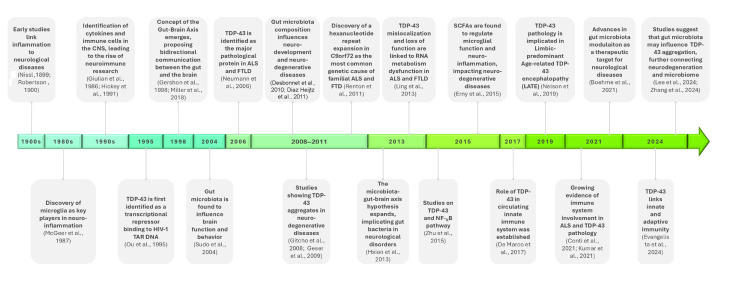
Key milestones in ALS research: Discoveries on TDP-43, immune responses, and the microbiome–gut–brain axis. ALS: Amyotrophic lateral sclerosis; FTLD: frontotemporal lobar degeneration; HIV-1 TAR DNA: human immunodeficiency virus type 1 TAR element; NF-κB: nuclear factor kappa-light-chain-enhancer of activated B cells; TDP-43: transactive response (TAR) DNA-binding protein 43.

TDP-43 aggregation lies at the intersection of several pathological processes in ALS, including immune dysfunction and microbiome alterations (**Figure 1**). This review explores the mechanisms connecting TDP-43 aggregation to neurodegeneration, with a focus on its role in NI, immune dysfunction, and microbiome interactions.

## Search Strategy

To provide a comprehensive understanding of the interplay between TDP-43, GM–brain axis, and immune responses in ALS, we conducted an extensive literature search to identify relevant *in vitro*, *in vivo*, and human studies. The primary aim was to explore the existing knowledge on these interconnected areas and evaluate current experimental models that examine their interactions. We utilized multiple databases, including PubMed, Google Scholar, Scopus, and Web of Science, to ensure a broad and targeted search. The search was focused using Medical Subject Headings (MeSH) to refine results and improve precision. Key MeSH terms relevant to the review topic were selected, including “TDP-43, amyotrophic lateral sclerosis, gut microbiota, immune system, brain, biological models, and gut-brain axis.” Boolean operators were applied to combine these MeSH terms effectively, such as: (“TDP-43” [Mesh]) AND (“amyotrophic lateral sclerosis” [Mesh]); (“TDP-43” [Mesh]) AND (“gut microbiota” [Mesh]) AND (“amyotrophic lateral sclerosis” [Mesh])); (“TDP-43” [Mesh]) AND (“immune system” [Mesh]) AND (“amyotrophic lateral sclerosis” [Mesh]); (“gut microbiota” [Mesh]) AND “brain” [Mesh]; (“immune system” [Mesh]) AND (“TDP-43” [Mesh]); (“TDP-43” [Mesh]) AND (“gut-brain axis” [Mesh]). We prioritized peer-reviewed articles with clearly described methods, relevant animal models or patient cohorts, and sufficient detail to assess validity of the outcome. Emphasis was placed on meta-analyses, systematic reviews, and both *in vitro* and *in vivo* studies, as these provide consolidated insights into current research trends. Reviews exploring future research trajectories and emerging therapeutic approaches were also incorporated to give a broader perspective on potential advancements in understanding the GM-brain-immunity axis in relation to TDP-43. This search strategy enabled a comprehensive review of current findings while ensuring a focused exploration of ALS-specific mechanisms, integrating diverse methodologies and perspectives on the evolving connections between TDP-43 pathology, gut microbiota, and immune dysregulation.

## Molecular Features and Functional Roles of TDP-43 in Amyotrophic Lateral Sclerosis

**Figure 2:**
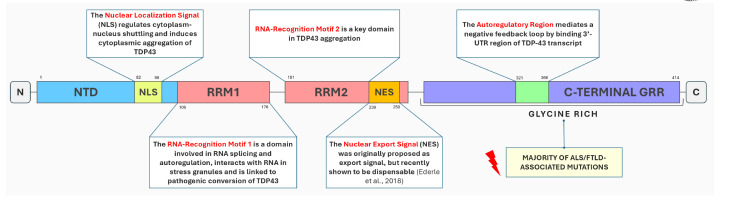
TDP-43 structure. TDP-43 shows many functional domains. Starting from the N-terminal domain, the protein exhibits a NLS followed by two RNA recognition motifs (RRM1 and RRM2). The C-terminal domain is characterized by the presence of a glycine rich region, and it represents the sequence with most of the disease-associated mutations. ALS: Amyotrophic lateral sclerosis; C: carboxy-terminus; FTLD: frontotemporal lobar degeneration; GRR: glycine rich region; N: amino-terminus; NES: nuclear export signal; NLS: nuclear localization signal; NTD: N-terminal domain; RRM-1 and -2: RNA-recognition motif 1 and 2; TDP43: transactive response (TAR) DNA-binding protein 43; UTR: untranslated region.

The evolutionary conservation of many domains of TDP-43 highlights its critical role, especially since its most conserved regions, RNA recognition motifs 1 (RRM1) and 2 (RRM2), are essential for its function (Wang et al., 2004; **Figure 2**).

**Figure 3:**
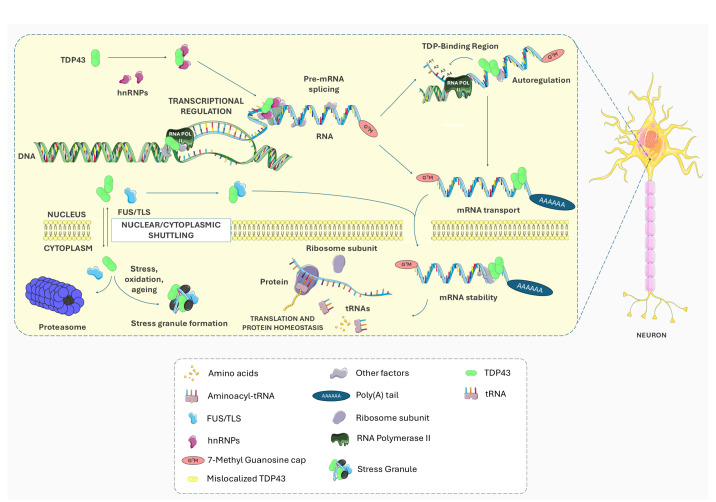
Schematic representation of TDP-43 cellular functions. TDP-43 has both nuclear and cytoplasmic functions. In the nucleus, TDP-43 usually collaborates with other proteins to modulate the RNA transcription, maturation, and transport. In the cytoplasm, TDP-43 has a role in mRNA stability, translation, degradation, and stress granule formation. Image provided by Servier Medical Art (https://smart.servier.com/), licensed under CC BY 4.0 (https://creativecommons.org/licenses/by/4.0/). hnRNP: Heterogeneous ribonucleoprotein particle; FUS/TLS: RNA-binding protein fused in sarcoma/translocated in liposarcoma; RNA Pol II: RNA polymerase type II complex; TDP43: transactive response (TAR) DNA-binding protein 43; tRNA: transfer RNA.

Subcellularly, TDP-43 is primarily localized in the nucleus, but several studies have demonstrated its continuous shuttling between the nucleus and cytoplasm, where it tends to form stable aggregates with other proteins and RNAs (Ayala et al., 2008; **Figure 3**). Furthermore, cytoplasmic inclusions of TDP-43 often hold pathological significance. In 2006, TDP-43 was identified as the major component of α-synuclein and tau-negative inclusions found in the brain and spinal cord of ALS (Neumann et al., 2006).

About its structure, TDP-43 shows the canonical heterogeneous ribonucleoprotein particle organization. In detail (**Figure 2**), we identify:

• N-terminal domain: It contains a conserved nuclear localization signal (NLS), divided into NLS1 and NLS2, essential for nuclear import. Mutations in these motifs decrease nuclear TDP-43 levels (Buratti and Baralle, 2001; Winton et al., 2008). Structurally, the N-terminal domain adopts a ubiquitin-like β-grasp fold, consisting of an α-helix packed against a β-sheet, forming a hydrophobic core nearly identical to ubiquitin despite low sequence similarity. A nuclear magnetic resonance study revealed this fold coexists with an unfolded state, with folding being favored by ssDNA binding (Qin et al., 2014).

• RNA recognition motifs (RRMs): The RRMs bind specific RNA/DNA sequences, including the 3′ UTR of *TARDBP* mRNA, contributing to autoregulation of TDP-43 levels (Ayala et al., 2011). A leucine-rich sequence in RRM2 (residues 239–250) was proposed as a canonical nuclear export signal (Strong et al., 2007), but later studies using mutagenesis, CRM1 inhibition, and live-cell imaging showed it is non-functional (Berson et al., 2017; Archbold et al., 2018; Chou et al., 2018; Ederle et al., 2018). Instead, nuclear export appears to occur mainly by passive diffusion. Meanwhile, the ubiquitin-like fold of N-terminal domain supports dimerization and higher-order assembly, crucial for splicing and nuclear retention (Mompeán et al., 2016; Afroz et al., 2017; Chou et al., 2018).

• C-terminal domain: It is intrinsically disordered and mediates protein–protein interactions via a glycine-rich region (Buratti et al., 2005) and a Q/N-rich prion-like domain. It harbors most ALS-associated mutations (Lagier-Tourenne et al., 2010), which alter solubility and promote pathogenic aggregation (Ayala et al., 2008). The C-terminal domain drives liquid–liquid phase separation via transient π–π and cation–π interactions, with a short α-helix (residues 321–330) acting as a nucleation site (Cao et al., 2019; Zhuo et al., 2020; Li et al., 2021), potentially underlying the strain-like behavior of TDP-43 aggregates in neurodegenerative diseases.

TDP-43 is essential for RNA metabolism, participating in transcription, splicing, transport in the nucleus, and regulating mRNA stability, translation, degradation, and stress granule formation in the cytoplasm (Buratti and Baralle, 2001, 2012; **Figure 3**). Under stress conditions, it can mislocalize to the cytoplasm forming inclusions, influenced by mutations and post-translational modifications, such as phosphorylation, ubiquitination, acetylation, SUMOylation, and cleavage (Budini et al., 2015; Morgan et al., 2017).

ALS-related mutations often cause nuclear depletion and cytoplasmic accumulation of TDP-43, starting with phosphorylated pre-inclusions that develop into aggregates also marked by ubiquitination (Lee et al., 2011). Despite its nuclear localization, TDP-43 constantly shuttles between nucleus and cytoplasm via NLS and nuclear export signal; alteration of this trafficking promotes aggregation (Winton et al., 2008; Igaz et al., 2011; Lee et al., 2011).

TDP-43 toxicity is explained by two models: “gain-of-function,” where aggregates impair autoregulation and promote cell death, and “loss-of-function,” where sequestration into stress granules leads to functional depletion. These mechanisms may coexist, accelerating disease (Igaz et al., 2011; Lee et al., 2011).

Post-translational modifications critically modulate TDP-43 function, localization, and aggregation. Phosphorylation at S409/410 enhances cytoplasmic mislocalization; proteolytic cleavage produces C-terminal fragments prone to aggregate; ubiquitination mirrors impaired clearance of misfolded TDP-43; acetylation at K145 disrupts RNA binding and promotes aggregation, while SUMOylation may influence solubility and trafficking (Buratti, 2018).

One of the aims of therapeutic strategies should be to act on the protein aggregation. Molecular chaperones, mainly the HSP70 family, play a pivotal role in modulating TDP-43 aggregation and phase separation, processes that are central to the pathology of neurodegenerative diseases like ALS. HSP70 has been shown to interact directly with TDP-43, maintaining it in a dynamic, liquid-like state and preventing its transition into pathological aggregates. HSP70 overexpression decreases the formation of hyperphosphorylated TDP-43 inclusions, highlighting its protective role against aggregation (Gu et al., 2021). Additionally, HSP70 chaperones RNA-free TDP-43 into anisotropic intranuclear liquid spherical shells, preventing its aggregation into amyloid-like structures. This chaperone activity is crucial in maintaining TDP-43 solubility and function, especially under stress conditions (Salado et al., 2014; Chen et al., 2020; Hans et al., 2020). Beyond HSP70, other chaperones like HSPB8, in collaboration with HSC70 and BAG3, facilitate the autophagic clearance of misfolded TDP-43, further emphasizing the relevance of chaperone-mediated pathways in mitigating TDP-43 toxicity (Mogk et al., 2019). Pharmacological strategies targeting these chaperone systems are being explored. For instance, enhancing autophagy through mTOR inhibitors like rapamycin has shown promise in promoting the degradation of TDP-43 aggregates (Buratti, 2021).

In summary, molecular chaperones are integral to the regulation of TDP-43 aggregation and phase behavior. Their modulation offers a promising avenue for therapeutic approaches in TDP-43-associated neurodegenerative disorders. Given the emerging evidence on the anti-aggregation effects of gut-derived microbial metabolites, as will be further explored in the following sections, future research should aim to investigate how GM modulation might synergize with existing anti-aggregation therapies. This integrative approach could offer novel strategies to target protein misfolding in ALS, highlighting the relevance of the gut-brain axis (GBA) in disease pathogenesis and treatment.

## TDP-43 and Gut Microbiota–Brain Axis

The GM plays a central role in the GBA communication, a bidirectional network of signals connecting the CNS and the enteric nervous system (ENS; Carabotti et al., 2015). Through the production of metabolites, modulation of cytokines’ release, and interaction with immune cells, the GM contributes to GBA signaling. However, the complex yet balanced interaction between the ENS and immune cells can be disrupted by dysbiosis (Menees et al., 2022), leading to increased intestinal permeability and systemic inflammation. Accumulating evidence suggests that GM alterations contribute to ALS pathophysiology. Both preclinical and clinical studies have identified significant changes in GM composition and function, supporting the notion that dysbiosis may modulate NI and ALS progression (Zhang et al., 2017; Figueroa-Romero et al., 2019; Burberry et al., 2020; Niccolai et al., 2021).

To provide a clearer overview of these findings, we have summarized the relevant studies into two complementary tables. **Table 1** outlines preclinical studies employing ALS models based on *C9orf72* or *TARDBP* mutations, alongside *in vitro* and *in vivo* investigations into the role of inflammation in TDP-43 pathology. These studies collectively highlight the impact of microbiota-targeted interventions, such as butyrate supplementation, probiotic administration, and fecal microbiota transplantation (FMT), on key pathological features of ALS. Reported outcomes include the restoration of intestinal barrier (IB) integrity, decrease of systemic inflammation and NI, improved neuromuscular performance, and reduced TDP-43 aggregation. Notably, one study demonstrates that pro-inflammatory stimuli can directly induce TDP-43 mislocalization and aggregation in both glial and neuronal cells, providing mechanistic insight into the inflammatory regulation of TDP-43 proteinopathy. These findings underscore the relevance of the gut–immune–brain axis in ALS and support the potential of microbiota-based strategies in modulating disease progression.

**Table 1:** Summary of preclinical studies examining the impact of gut microbiota on ALS, based on transgenic mouse models carrying *C9orf72* or *TARDBP* mutations, as well as *in vitro* and *in vivo* models of TDP-43 proteinopathy

Type of study	Key finding	Reference
Preclinical experimental study in C9orf72 ALS mice.	Low-immune-stimulation environments reduced systemic inflammation and prolonged survival. Broad-spectrum antibiotics and gut microbiota transplants from a protective environment mitigated inflammatory responses, even post-symptom onset, underscoring microbiota-immune interactions in ALS progression.	Burberry et al., 2020
Interventional study in TDP-43 A315T transgenic mice	TDP-43 mice exhibited early gut dysfunction, reduced intestinal motility, increased permeability, and microbial dysbiosis (↓ butyrate producers, ↑ Bacteroides fragilis). Systemic inflammation and TDP-43 aggregation were elevated. Treatment with butyrate or the probiotic VSL#3 restored gut barrier integrity, reduced inflammation and protein aggregation, and improved neuromuscular performance. Findings support the gut–brain axis as a therapeutic target in ALS.	Zhang et al., 2024
Experimental preclinical study involving both *in vitro* and *in vivo* models	The study shows that inflammatory stimuli (LPS, TNF-α) induce TDP-43 cytoplasmic mislocalization and aggregation in glial and motor neuron-like cells. *In vivo*, chronic inflammation worsens TDP-43 pathology in transgenic mice, suggesting a direct role of neuroinflammation in TDP-43 proteinopathy.	Correia et al., 2015

These studies assess the effects of microbiota-targeted interventions, such as fecal microbiota transplantation, butyrate supplementation, and probiotics, on key pathological features, including neuroinflammation, intestinal barrier integrity, TDP-43 aggregation, and neuromuscular function. Overall, the findings underscore the involvement of the gut–immune–brain axis in ALS and support microbiota-based approaches as potential disease-modifying strategies. ALS: Amyotrophic lateral sclerosis; LPS: lipopolysaccharide; TDP-43: TAR DNA-binding protein 43; TNF-α: tumor necrosis factor-alpha.

**Table 2** complements these findings with clinical and observational studies in ALS patients. These reports frequently document gut dysbiosis, including reduced microbial diversity, depletion of short-chain fatty acid (SCFA)-producing bacteria, and alterations in the Firmicutes/Bacteroidetes ratio. Some studies also correlate microbial shifts with systemic inflammatory profiles or specific clinical phenotypes, such as bulbar or spinal onset. Where available, we have indicated the potential relevance to TDP-43 pathology based on genetic background, animal model, or clinical presentation.

**Table 2:** Overview of human studies analyzing GM profiles in ALS patients compared to healthy controls

Type of study	Key finding	TDP-43 relevance	Reference
Case-control study comparing GM and fecal metabolites in eight ALS patients and eight healthy controls.	ALS patients had a higher abundance of Firmicutes and a significant reduction in Faecalibacterium and Bacteroides. SCFA, nitrite/nitrate (NO₂-N/NO₃-N), and GABA levels were elevated, suggesting metabolic alterations in ALS.	TDP-43 relevance not assessed; no genetic or pathological data provided.	Zhai et al., 2019
Case-control studies analyzing GM differences in ALS patients.	ALS patients showed reduced microbial diversity, depletion of beneficial bacteria (Oscillibacter, Anaerostipes, Lachnospiraceae, and Ruminococcus), and an increase in harmful genera (Dorea), indicating dysbiosis and potential gut-brain axis involvement in ALS pathogenesis.	Likely TDP-43-related (sporadic ALS cohort; no genetic testing reported).	Fang et al., 2016; Rowin et al., 2017
Preclinical experimental study on microbiota in ALS.	Mazzini et al. reported reduced gut microbial diversity and tested Lactobacillus-based probiotics, showing potential for improving gut barrier integrity.	Presumed TDP-43 pathology (sporadic ALS model; no mutation-specific data).	Mazzini et al., 2018
Longitudinal clinical study on microbiota in ALS.	Di Gioia et al. found distinct GM profiles in 50 ALS patients, with microbial diversity declining over 6 mon; probiotic supplementation did not affect disease progression.	Likely TDP-43 pathology (sporadic ALS patients; no genetic screening performed).	Di Gioia et al., 2020
Case-control study comparing GM and fecal metabolites in 20 ALS patients and 20 healthy controls.	ALS patients had increased Bacteroidetes and related genera, with decreased Firmicutes and Megamonas. Metagenomic analysis showed reduced gene functions linked to key metabolic pathways, suggesting a role of microbial metabolism in ALS pathogenesis.	Presumed TDP-43-related (based on sporadic ALS cohort; lacks genetic/molecular confirmation).	Zeng et al., 2020
Case-control study on butyrate-producing bacteria in ALS patients.	ALS patients had significantly lower levels of butyrate-producing bacteria (Eubacterium rectale and Roseburia intestinalis) even after adjusting for age, sex, and constipation. The loss of eight dominant butyrate producers suggests a role of GM alterations in ALS progression.	Consistent with TDP-43 pathology (sporadic ALS; no genetic data, but clinical presentation typical).	Nicholson et al., 2021
Study comparing inflammatory and microbiota profiles in ALS patients with different clinical characteristics, compared to healthy family caregivers	Inflammatory cytokine profiles differed between ALS patients and controls, as well as between patients with different disease progression rates. 16S metagenome analysis revealed a reduction in butyrate-producing bacteria and a decrease in the Firmicutes/Bacteroidetes ratio in ALS patients. The inflammatory cytokine and microbiota profiles may serve as potential biomarkers for ALS heterogeneity.	Presumed TDP-43-associated ALS (sporadic cases; no genetic or proteinopathy-specific data).	Niccolai et al., 2021
Observational case-control study comparing ALS patients and their spouses.	Spinal-onset ALS patients exhibited gut dysbiosis with an increased Firmicutes/Bacteroidetes (F/B) ratio, while bulbar-onset ALS patients showed oral dysbiosis with a decreased oral F/B ratio. Both ALS subtypes displayed microbial translocation associated with more severe symptoms, suggesting oral dysbiosis may be a pathogenic factor in bulbar ALS.	Likely TDP-43 pathology, particularly in bulbar-onset ALS (bulbar phenotype strongly associated with TDP-43).	Kim et al., 2022
Mendelian Randomization study assessing causal links between GM and ALS risk.	Higher levels of Enterobacteriaceae and unclassified Acidaminococcaceae species were associated with increased ALS risk. A genetically predicted ALS risk was linked to increased OTU4607_Sutterella and Lactobacillales_ORDER abundance, suggesting a bidirectional relationship between GM and ALS.	Probable TDP-43 involvement (based on Genome-Wide Association Studies data from predominantly sporadic ALS cohorts).	Zhang et al., 2022

The table includes case-control, longitudinal, and Mendelian randomization studies. Despite methodological variability, most investigations report reduced microbial diversity and a depletion of butyrate-producing bacteria in ALS. Several studies also associate GM alterations with disease progression or clinical phenotype, highlighting potential microbiota-based biomarkers and mechanistic pathways involved in ALS pathophysiology. To mirror the focus of this review, an additional column has been included to indicate the relevance of TDP-43 pathology, based on clinical, genetic, or pathological information when available. ALS: Amyotrophic lateral sclerosis; GABA: gamma-aminobutyric acid; GM: gut microbiota; SCFAs: short-chain fatty acids; TDP-43: TAR DNA-binding protein 43.

Taken together, these studies support the hypothesis that altered GM composition and/or function can influence ALS progression by impairing both IB and blood–brain barrier (BBB) integrity, which are critical anatomical regulators of communication between the gut and brain (Menees et al., 2022). Therefore, preserving the integrity of the IB and BBB is critical: the immune system (IS) plays a crucial role in this process, by protecting against harmful pathogens and NI while ensuring effective GBA communication, thereby safeguarding both gut and overall health.

While **Tables 1** and **2** summarize key findings, a critical evaluation of methodological quality is essential to contextualize the evidence. Over the past decade, animal and human studies have advanced our GM understanding in ALS, though substantial methodological heterogeneity limits interpretability.

Preclinical studies in transgenic mice (e.g., C9orf72, TDP-43 models) link gut dysbiosis to immune activation and disease progression. Interventions like FMT, butyrate, and probiotics show benefits on barrier integrity, inflammation, motor function, and TDP-43 aggregation (Burberry et al., 2020; Zhang et al., 2024). However, species differences and simplified models limit the translational relevance. Clinical studies consistently report reduced microbial diversity and depletion of SCFA-producing taxa in ALS patients (Zhai et al., 2019; Di Gioia et al., 2020; Nicholson et al., 2021). Emerging data suggest phenotype-specific microbial signatures (Kim et al., 2022) yet studies are hindered by small samples, limited genetic/pathological profiling, and poor control for confounders. Collectively, evidence supports a role for the GM–immune–brain axis in ALS. Future research should integrate multi-omics and genetic stratification, ideally with post-mortem validation of TDP-43 pathology, to better align mechanistic insights with clinical relevance.

Having considered these methodological limitations, we now turn to the mechanistic pathways of the GBA which may contribute to ALS pathophysiology. In this regard, metabolic pathways are integral to the core mechanisms enabling an interdependence between the gut and the CNS and metabolic function has been recently studied as being influenced by GM, apart from local and/or systemic immunity. Indeed, metabolic dysregulation is now increasingly recognized as a core component of ALS pathophysiology, occurring at both the systemic and cellular levels, and intersecting with IS dysfunction and neurodegenerative processes (Gautam et al., 2025). Emerging evidence also implicates alterations in the GM as a contributing factor to these metabolic disturbances. Such changes can influence host metabolism by compromising gut barrier function, promoting systemic inflammation, and directly affecting energy regulation (Martin et al., 2019).

Notably, the gut dysbiosis has been linked to reduced efficiency in nutrient absorption, which may help explain the hypermetabolic condition frequently seen in ALS patients. This state is marked by elevated resting energy expenditure, progressive weight loss, and muscle atrophy, even in the presence of normal or increased caloric intake (Vaisman et al., 2009; Fayemendy et al., 2021; Corbin et al., 2023).

On a cellular scale, accumulating evidence highlights the role of TDP-43 proteinopathy as a key contributor to metabolic disturbances. Multiple mitochondrial pathways have been shown to be affected by TDP-43 dysfunction in animal models, where genetic or dietary interventions targeting these metabolic routes alleviated TDP-43-dependent locomotor impairments (Loganathan et al., 2022). Recent findings suggest that the cytoplasmic mislocalization of endogenous TDP-43, independent of its nuclear loss-of-function, can start early metabolic disruptions that mimic those observed at the ALS onset. In detail, Hu et al. conducted a trascriptomic analysis in a zebrafish cell line engineered to express cytoplasmically mislocalized TDP-43, identifying significant dysregulation in genes involved in energy metabolism. These results support the hypothesis that TDP-43 mislocalization leads to cytoplasmic gain-of-function effects, contributing to metabolic imbalances during the early phases of disease progression (Hu et al., 2024).

At the systemic levels, clinical studies have shown that increased plasma levels of adiponectin in ALS patients may drive TDP-43 mislocalization in both peripheral blood mononuclear cells (PBMCs) and MNs. This process appears to be mediated by AMPK activation and may play a role in the disrupted systemic energy homeostasis observed in ALS (Liu et al., 2024).

**Figure 4:**
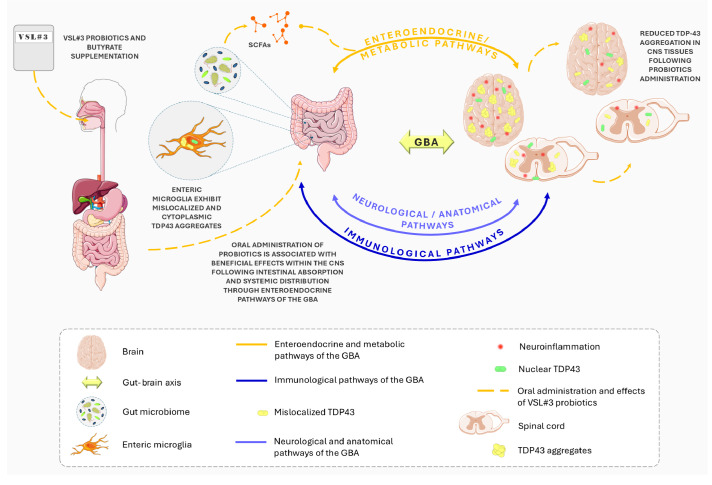
Bidirectional gut–brain communication and the role of probiotics and butyrate in TDP-43 pathology in ALS. This figure illustrates the bidirectional communication between the gut and the CNS, known as the gut-brain axis, mediated by enteroendocrine/metabolic, neurological/anatomical, and immunological pathways. The gut microbiome influences CNS function through bioactive metabolites like SCFAs. The zoomed-in gut sections highlight the role of enteric glia in TDP-43 pathology, reflecting processes seen in the CNS. A study in TDP-43 transgenic mice (Zhang et al., 2024) shows that butyrate and probiotics, such as VSL#3, significantly slow disease progression by reducing TDP-43 aggregation and glial fibrillary acidic protein expression, while enhancing intestinal barrier and blood-brain barrier integrity markers (α-smooth muscle actin, zonula occludens-1, and claudin-5). Image provided by Servier Medical Art (https://smart.servier.com/), licensed under CC BY 4.0 (https://creativecommons.org/licenses/by/4.0/). ALS: Amyotrophic lateral sclerosis; CNS: central nervous system; GBA: gut–brain axis; SCFAs: short-chain fatty acids; TDP-43: transactive response (TAR) DNA-binding protein 43.

Immune activation, another key ALS feature is also tightly intertwined with metabolic cues. Immune cell phenotypes and effector functions are critically regulated by their metabolic state, and several GM metabolites can influence the immunometabolic landscape. For instance, SCFAs, such as butyrate and propionate, modulate mitochondrial function and lipid metabolism, shaping both peripheral immune responses and CNS homeostasis (Hu et al., 2018; Kassan et al., 2023). These microbial metabolites have been shown to exert neuroprotective effects in ALS models, in part by attenuating the accumulation of pathological proteins, including TDP-43, and diminishing inflammatory responses in glial cells (Zhang et al., 2017; Caetano-Silva et al., 2023; Sun and Zhang, 2024). The production of SCFAs from fermentation of dietary soluble fibers and starch stands out as one of the major ways through which GM contributes to intestinal and systemic health (Portincasa et al., 2022; **Figure 4**), alongside the production of tryptophan derivatives (Gupta et al., 2023) and bile acids (Collins et al., 2023), which can be all circulated via the lymphatic and systemic circulation (Cummings et al., 1987; Yu et al., 2022), eventually affecting other organ systems and tissues such as BBB. The functional GM study has led to the understanding of how the different ratios of such metabolites can give an account to overall health. A balanced microbiome is, in fact, not only a relevant organ for the proper digestion function of complex nutrients, but it is essential as a protective barrier from pathogens as well as a shaper of immunity. Intestinal SCFAs exert local and systemic effects as they are absorbed and circulated (Kien et al., 2008; Peng et al., 2009; Lewis et al., 2010). Indeed, after absorption, either directly or following liver metabolism (e.g., propionate; Kim, 2024), SCFAs travel throughout the body and act as signaling molecules by interacting with G-protein-coupled receptors expressed in several tissues (Kim et al., 2013; Masui et al., 2013), including the CNS tissues (Nøhr et al., 2015). Here, SCFAs regulate tight junction proteins, which are essential for the maintenance of BBB integrity, regulate inflammation and stimulate the proper expression of neurotrophic factors, including nerve growth factor, glial cell line-derived neurotrophic factor, and brain-derived neurotrophic factor (Braniste et al., 2014; Barichello et al., 2015; Wenzel et al., 2020). SCFAs can shape the phenotype of microglia, modulate neurotransmitters production, regulate intracellular pathways underlying NI, and influence the BBB integrity (Colombo et al., 2021; Tang et al., 2022; Caetano-Silva et al., 2023). The GM role in regulating BBB function has been investigated in germ free mice, where some studies led to the observation that such mice exhibit increased BBB permeability, which is associated with reduced expression of endothelial tight junction proteins occludin and claudin-5, compared to mice with a healthy GM (Braniste et al., 2014).

Additional evidence suggests that GM dysbiosis may influence neurodegenerative processes through its impact on TDP-43 aggregation and immune modulation (Figueroa-Romero et al., 2019; Zhang et al., 2024). ALS mouse models show that GM alterations increase intestinal permeability, promote systemic inflammation and accelerate TDP-43 mislocalization in the CNS (Wu et al., 2015; Rowin et al., 2017; Lee et al., 2024; Zhang et al., 2024). Such consequences suggest that gut dysregulation may occur early in ALS process, potentially preceding neuromuscular symptoms. In fact, alterations in the gut permeability can lead to local or systemic exposure to microbial metabolites and products such as endotoxins. LPS, for instance, can act on distal tissues like the CNS and impact microglia activation (Dai et al., 2015; Bowyer et al., 2020). These bacterial products can translocate through the IB to enter the circulatory system, causing metabolic endotoxemia and eventually activation of immune responses and NI within the brain (Alhasson et al., 2017).

There has been growing evidence suggesting that elevated LPS levels in the bloodstream of ALS patients may indicate that gut inflammation and GM alterations could contribute to the ALS development (Zhang et al., 2009). Furthermore, the LPS addition to microglia or astrocytes *in vitro* leads to improper localization and aggregation of TDP-43 (Correia et al., 2015), whose aggregates are detected in the majority of ALS cases.

**Figure 5:**
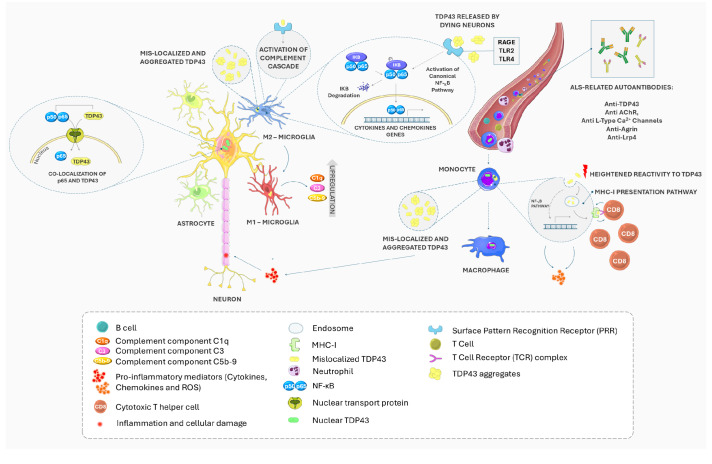
Role of TDP-43 in immune cell functions within ALS context. TDP-43 aggregates, released by dying neurons, act as damage-associated molecular patterns, triggering immune responses. These aggregates initiate the activation of PRRs and NF-κB signaling pathways in glial cells, subsequently inducing the expression of pro-inflammatory genes. This cascade drives the transition of microglia from a neuroprotective M2-like phenotype during the early stages of ALS to a neurotoxic M1-like state as the disease progresses. The shift to the M1 phenotype results in ROS production, the release of cytokines and chemokines, and further recruitment of leukocytes to the central nervous system, exacerbating the neuroinflammatory response. As shown in the zoomed-in within microglial cells, TDP-43 has been implicated in the activation of the microglial complement pathway. Studies have observed significant upregulation of complement components such as C1q, C3, and C5B-9, which are believed to contribute to microglial activation and enhanced synaptic pruning, a key feature of ALS pathogenesis. On the left side, the NF-κB signaling pathway is highlighted as a critical component in TDP-43-mediated neuroinflammation. Research indicates that TDP-43 and NF-κB share a common nuclear transport mechanism and TDP-43 overexpression results in the constitutive inhibition of NF-κB signaling. Additionally, studies in ALS models and patient tissues have demonstrated colocalization of TDP-43 and the p65 subunit of NF-κB within the nuclei of affected neurons. These findings suggest that TDP-43 may influence ALS pathogenesis in peripheral immune cells, where NF-κB signaling is crucial for their function. Further evidence indicates that ALS patients exhibit the typical mislocalization and aggregation of TDP-43 even in monocytes, with heightened reactivity of APC to TDP-43, responding to its internalization by upregulating inflammatory cytokines and presenting TDP-43-derived peptides via MHC molecules to CD8^+^ T cells. Finally, given the detection of ALS-related autoantibodies in patient sera, it has been suggested that TDP-43 could serve as an antigen driving an adaptive autoimmune response, as illustrated in the upper-right section of the figure. Image provided by Servier Medical Art (https://smart.servier.com/), licensed under CC BY 4.0 (https://creativecommons.org/licenses/by/4.0/). AChR: Acetylcholine receptor; C1q: complement component 1q; C3: complement component 3; C5b-9: complement component 5b-9; IKB: inhibitor of nuclear factor kappa B kinase; IL-1β: interleukin-1 Beta; Lrp4: low density lipoprotein receptor related protein 4; MHC-I: major histocompatibility complex type I; NF-κB: nuclear factor kappa-light-chain-enhancer of activated B Cells; p50: p50 subunit of NF-κB; p65: p65 subunit of NF-κB; RAGE: receptor for advanced glycation endproducts; ROS: reactive oxygen species; TNF-α: tumor necrosis factor alpha; TLR2 and TLR4: Toll-like receptors 2 and 4; TDP43: transactive response (TAR) DNA-binding protein 43.

The GM effects on TDP-43 aggregation have been investigated in animal models (Zhang et al., 2024). TDP-43 transgenic mice expressing the human mutated TDP-43 (hmTDP-43), express human mutant forms of the *TDP-43* gene primarily expressed in the brain and spinal cord and develop motor dysfunction by 6 months of age (Gordon et al., 2019) and L5 motor axons loss by 10 months (Arnold et al., 2013). Results from studies on such TDP-43 mouse models showed changes in the TDP-43 expression in the ENS and aggregation within both CNS and ENS (Guo et al., 2012; Esmaeili et al., 2013; Hatzipetros et al., 2014; Mitra et al., 2024; Zhang et al., 2024), an integral GBA part which might be then involved in gastrointestinal symptoms of ALS patients. Treatment of these mice with butyrate and probiotics significantly slowed disease progression (Zhang et al., 2024). This was achieved by reducing pathological hmTDP-43 aggregation and glial fibrillary acid protein expression while enhancing markers of IB and BBB integrity, including alpha-smooth muscle actin, ZO-1, and claudin-5 both in the colon and in the CNS (Zhang et al., 2024). Additionally, reduced hmTDP-43 aggregation was observed in enteric glial cells, highlighting the beneficial effects of butyrate and probiotic on gut and brain homeostasis (Zhang et al., 2024; **Figure 5**). Notably, the study also revealed that probiotic treatment improved intestinal motility, permeability, and barrier function, while reducing inflammatory cytokine levels and hmTDP-43 aggregation in the colon, spinal cord, and brain. This was accompanied by improved motor function, as evidenced by increased rotarod time in treated mice compared to untreated controls. The findings from Zhang et al. (2024) suggest that endotoxemia may contribute to acceleration of TDP-43 pathogenesis, while probiotic treatment appears to mitigate these effects, potentially by enhancing gut and brain integrity via the GBA. These results support the hypothesis that modulation of the GM and its metabolites, such as SCFAs, could represent a promising avenue for further research aimed at slowing ALS progression through anti-inflammatory actions and restoration of GBA homeostasis.

Together, these results further highlight the critical role of the GM-immunity axis in ALS pathogenesis, suggesting that disruptions in GBA communication may contribute to disease onset and progression. However, intestinal immunity and early gastrointestinal alterations in ALS remain underexplored, even though gastrointestinal symptoms often precede neurological ones in ALS patients (Wu et al., 2015). Emerging therapeutic approaches, such as FMT and dietary interventions based on a more integrative approach that considers the metabolic-immune-microbiome triad in relation to TDP-43 pathology, are essential to develop clinically meaningful strategies for ALS, offering potential and promising avenues for treating neurological and neurodegenerative disorders including ALS.

## TDP-43, Innate Immune Responses, and Neuroinflammation

ALS has largely been recognized as a neuroinflammatory condition where NI has been associated with disease progression and neuronal loss even during the presymptomatic disease phase (Thonhoff et al., 2018), since a significant correlation between the intensity of microglial activation in the motor cortex and the severity of clinical MN deficits has been demonstrated (Turner et al., 2004). While the right ALS etiopathogenesis remains unclear, it is well recognized as a complex, multifactorial disease in which immune mechanisms play a significant role (Beers et al., 2008; Finkelstein et al., 2011; Jin et al., 2020). Studies in both ALS patients and animal models have shown that changes in innate and adaptive immune cell populations can impact disease onset and progression (Butovsky et al., 2012; Lam et al., 2016; Zondler et al., 2016, 2017).

The innate IS is a central player in the pathogenesis of neurodegenerative diseases like ALS (Novellino et al., 2020), exhibiting a dual nature that alternates between neuroprotection and neurotoxicity (Liu et al., 2009; Zhao et al., 2010; Vinet et al., 2012; Shastri et al., 2013). The intricate interplay among cellular and molecular components of the innate IS underscores the necessity for precise spatial and temporal regulation to balance protective and detrimental effects in ALS. Although TDP-43 dynamics have mainly been studied within the nervous tissues, recent studies have explored its functions in immune cell populations, establishing a strong link between TDP-43 pathology and immune dysfunction through several mechanisms (Evangelista et al., 2024; **Figure 5**). Recent evidence suggests that TDP-43 aggregates act as damage-associated molecular patterns (DAMPs) (Evangelista et al., 2024), capable of activating and engaging further immune responses after the induction of interleukin-1 beta and interleukin-18 secretion via NOD-like receptors (NLRs), pyrin domain-containing protein 3 (NLRP3) and other pattern recognition receptor activation in glial cells, which consequently leads to NI (Zhao et al., 2015; Lee et al., 2020; Evangelista et al., 2023; **Figure 5**).

Glial cells, specifically microglia, are the principal innate immune cells of the CNS and are critical for neuronal development and maturation (Dixon et al., 2021). In their resting state, they exhibit a highly branched morphology, constantly surveying their environment and performing functions such as complement-dependent synaptic pruning, phagocytosis of apoptotic cells (Changeux and Danchin, 1976; Scott et al., 2001; Gilchrist et al., 2020), and supporting synaptic plasticity through the secretion of neurotrophic factors (Komori et al., 2024). A key feature of these cells is their ability to activate in response to specific pattern recognition receptor ligands (Olson and Miller, 2004), including Toll-like receptors (TLRs) and inflammasomes, through which they are able to recognize specific DAMPs such as soluble fibrillary Aβ, α-synuclein and TDP-43 aggregates, which are released by dying neurons (Reed-Geaghan et al., 2009; Zhao et al., 2010, 2015; Béraud and Maguire-Zeiss, 2012; LaRocca et al., 2019; Deora et al., 2020; Hutchings et al., 2024). The binding of these DAMPs to receptors such as receptor for advanced glycation endproducts, NLRP3, TLR2, and TLR4 triggers the nuclear factor kappa B (NF-κB) signaling pathway (Zhao et al., 2010; Wang et al., 2020; Dutta et al., 2021; MacLean et al., 2021), promoting the downstream expression of pro-inflammatory genes and the activation of adaptive immunity after the secretion of cytokines/chemokines, further perpetuating NI (Saijo et al., 2013). These events are characterized by a typical glial transition from a neuroprotective M2-like condition in the early disease stages to a neurotoxic M1-like phenotype as it progresses (Liao et al., 2012), a shift that orchestrates the neuroinflammatory response and exacerbates MN degeneration (**Figure 5**). Such microglial activation facilitates the release of cytokines/chemokines, promoting a further recruitment of leukocytes to the CNS and promoting a cascade of heightened inflammatory events (Zhou et al., 2006). Under health conditions, this transient inflammatory response is tightly regulated, with repair mechanisms counteracting inflammation to promote tissue healing and restore function (DiSabato et al., 2016; Dai et al., 2021). However, when this delicate balance is disrupted, NI can become chronic and harmful. In such cases, there is an overproduction of cytokines/chemokines, infiltration of peripheral immune cells, edema development, and increased BBB permeability. This cascade fosters prolonged inflammation, which can become self-sustaining and challenging to resolve (Larochelle et al., 2011; Rajesh and Kanneganti, 2022; Müller et al., 2025). Glial activation and NI emerge early and persist throughout ALS progression, with astrocytes playing a pivotal role in mediating the inflammatory processes that contribute to MN degeneration, which appears to be linked to mutations in astrocytic TDP-43 (Whalley, 2013; Stoklund Dittlau et al., 2023). Similar to microglia, in ALS astrocytes shift towards a reactive phenotype through a phenomenon known as astrogliosis, a transformation that is likely triggered by signals released by dying neurons, such as micro-RNAs, and is thought to occur downstream of microglial activation, which appears to anticipate astrocyte reactivity (Brites, 2012; Pascual et al., 2012; Soubannier et al., 2024). In fact, experiments on ALS mouse models have demonstrated that *SOD1* mutations are associated with a neurotoxic A1-like phenotype that has negative consequences on MN health following the release of inflammatory factors (Liddelow et al., 2017).

During the last decade, the crosstalk between astrocytes and the IS has been studied in more detail, and it has been established an association between astrogliosis and the phenotypic shift previously described for microglial cells, since astrocytes appear to be critical regulators of microglial activation and NI in ALS (Ouali Alami et al., 2018). This modulation appears to be stage-dependent, as documented in animal models, where astrocytic activation of NF-κB evolves with disease progression and contributes to microglial proliferation and immune cell infiltration within the CNS (Ouali Alami et al., 2018). In this scenario, astrocytes could be seen as the joining link between microglial functions and adaptative cell-mediated immune responses, an interaction that stands out as a crucial point of communication that drives the CNS microenvironment toward a pro-inflammatory state, ultimately worsening neuronal damage. In fact, increasing evidence highlights a dynamic, stage-dependent crosstalk among microglia, astrocytes, and infiltrating immune cells in ALS. This interplay appears to be associated with TDP-43 neuronal aggregates and astrocytic mutations, which respectively promote a dysfunctional immune profile in microglia and a neurotoxic phenotype in astrocytes (Whalley, 2013; Swanson et al., 2025). Intercellular signaling plays a key role in this process: microglia release pro-inflammatory factors such as TNF-α and C1q, which drive astrocytes toward a harmful A1 phenotype (Liddelow et al., 2017). In turn, astrocyte-derived transforming growth factor beta 1 influences microglial activation – biasing it toward a detrimental state – and restricts T cell infiltration, thus creating a self-perpetuating inflammatory loop that exacerbates inflammation and accelerates MN degeneration (Endo et al., 2015).

## Molecular Crosstalk between TDP-43 and Systemic Immunity

The link between immunity, inflammation, TDP-43 pathology, and ALS is further supported by the fact that numerous genes modulated by TDP-43 are highly expressed in innate immune and inflammatory pathways. This suggests that TDP-43-driven immune dysregulation plays a fundamental role in disease progression. Among these genes, *C9orf72* is known to be involved in autoimmunity, systemic inflammation, and NI (Nataf and Pays, 2015; O’Rourke et al., 2016; Rizzu et al., 2016; Sakkas et al., 2017). Within innate immune cells such as dendritic cells and microglia, *C9orf72* is crucial for maintaining immune homeostasis, and its dysregulation has been linked to a heightened immunity and chronic microglial activation (Atanasio et al., 2016; O’Rourke et al., 2016; Lorenzini et al., 2020; Masrori et al., 2022). Similarly, TANK-binding kinase 1 gene (*TBK1*) encodes a serine/threonine kinase that regulates NF-κB signaling in response to TNF-α within innate immune cells (Gao et al., 2022; Runde et al., 2022). *TBK1* mutations have been associated with TDP-43 proteinopathy in ALS (Hao et al., 2021), where they lead to reduced activation of interferon regulatory factor 3, a key transcription factor in innate immunity (Ahmad et al., 2016), which regulates LPS-mediated neuroinflammatory responses (Joshi et al., 2024).

Among the inflammatory pathways consistently associated with TDP-43, NF-κB signaling stands out as particularly significant (Swarup et al., 2011; Zhao et al., 2015). NF-κB functions as a transcription factor that regulates the expression of hundreds of genes involved in inflammation and innate immune responses. Interestingly, TDP-43 and NF-κB share the same classic nuclear transport pathway (Zhu et al., 2015), suggesting that they may functionally interact by competing for nuclear transport machinery (Zhu et al., 2015; **Figure 5**). Supporting this, experimental studies have documented that TDP-43 overexpression leads to constitutive inhibition of NF-κB signaling (Zhu et al., 2015). In neurodegenerative diseases such as ALS, TDP-43 has been observed colocalizing with the NF-κB subunit p65 in the nuclei of neurons from ALS patients (Bright et al., 2021), as well as in transgenic mice expressing human wild-type and mutant TDP-43 (Swarup et al., 2011; Bright et al., 2021). Furthermore, elevated mRNA and protein levels of both TDP-43 and NF-κB have been reported in the spinal cords of ALS patients (Julien et al., 2012), and experimental exposure to inflammatory stimuli such as TNF-α or LPS, which activate NF-κB, has been shown to exacerbate TDP-43 proteinopathy in various cultured cells, including microglia, astrocytes, and neurons (Correia et al., 2015). This phenomenon was further corroborated *in vivo*, where LPS administration in TDP-43^A315T^ transgenic mice led to increased cytoplasmic TDP-43 accumulation and aggregation in spinal MNs (Correia et al., 2015).

Given this evidence, TDP-43 accumulation may also contribute to ALS pathogenesis in circulating white blood cells and humoral immunity, beyond the CNS. De Marco et al. (2017) investigated the TDP-43 accumulation in circulating monocytes of ALS patients carrying mutant *TARDBP*, aligning with similar findings in MNs and microglia. Monocytes play a crucial role in neurodegeneration by infiltrating the CNS through a compromised BBB (Liu et al., 2020). Upon entering the CNS, they differentiate into macrophages, contributing to the inflammatory milieu (Zhang et al., 2025). Once locally polarized toward an M1-like phenotype, infiltrating monocytes exhibit altered adhesion properties and impaired phagocytosis, further implicating them in ALS pathology (Liu and Wang, 2017). However, the appropriate mechanisms through which TDP-43 affects monocyte behavior remain to be elucidated.

Microglia are equipped with a variety of phagocytic receptors, including complement receptors that recognize synapses tagged for remotion (Wakselman et al., 2008; Zhang et al., 2014). The complement system modulates microglial activity, and its chronic activation has been linked to TDP-43 pathology (Lee et al., 2018). In ALS, complement proteins such as C1q, C4, and the terminal complement complex C5b-9 have been found upregulated in microglial cells, cerebrospinal fluid, CNS tissues, and serum of ALS patients, suggesting a pathogenetic contribution to the disease (Tsuboi and Yamada, 1994; Sta et al., 2011; Mantovani et al., 2014). Increased levels of these complement components correlate with microglial activation and MN damage, and the C5a-C5aR1 signaling axis is emerging as a promising therapeutic target to mitigate NI (Lee et al., 2017; Zhu et al., 2025). Notably, in TDP-43^Q331K^ mouse model of ALS, C5aR1 is upregulated, contributing to MN degeneration and neuromuscular junction disruption (Lee et al., 2018), and imbalanced local complement activation exacerbates MN damage in ALS patients (Kjældgaard et al., 2018, 2021). Proteomic analysis of TDP-43 mutants revealed significant upregulation of complement components, including C1q, C3, and C5b-9, which are believed to mediate microglial reactivity and enhance synaptic pruning (Wei et al., 2023; **Figure 5**). Specifically, Wei et al. (2023) revealed that TDP-43 activates the microglial complement pathway, resulting in motor dysfunction. Interestingly, exercise-induced upregulation of clusterin has been shown to inhibit complement cascade activation, offering a potential strategy for mitigating the neuroinflammatory damage seen in ALS patients.

## TDP-43 and Adaptive Immune Responses

The complex crosstalk among these cellular and molecular players within the innate IS forms an intricate network that underlies ALS pathophysiology. Given this evidence, TDP-43 pathology extends beyond neuronal toxicity to include significant immune dysregulation, engaging innate and adaptive immunity, lastly contributing to ALS onset and progression. In fact, TDP-43 aggregates can be internalized by antigen-presenting cells (APCs) such as macrophages and microglia, leading to vesicle rupture, release of pro-inflammatory signals, and general immune activation (Paz et al., 2010; Jiang et al., 2017; **Figure 5**). Peripheral immune cells from ALS patients exhibit heightened reactivity to TDP-43, implicating its role in adaptive immunity, and transcriptomic studies reveal upregulation of cytokines’ production in response to TDP-43 stimulation, further supporting this evidence. Once phagocytosed, TDP-43 is processed and presented via major histocompatibility complex (MHC) molecules, leading to T cell activation, thereby linking innate and adaptive immunity in ALS. Imaging studies conducted in ALS brain tissue reveal APC interactions with CD8^+^ memory T cells, indicating ongoing antigen presentation. *In vitro* studies further confirm that TDP-43 induces immune synapse formation, since the stimulation of co-culture models with TDP-43 leads to an induction of immune synapse formation between APCs and naïve T cells, finally leading to T cell activation (Evangelista et al., 2024).

CD8^+^ T-cells have been observed in the brain and spinal cord of both ALS patients and mouse models (Engelhardt et al., 1993; Fiala et al., 2010; Coque et al., 2019), and *in vivo* studies not only revealed that these cells infiltrate CNS at the symptomatic stage, but also that the amount of T cell infiltrates within the CNS increases with disease progression (Beers et al., 2008; Chiu et al., 2008). Moreover, some studies have observed increased counts of CD8^+^ T cells in the peripheral blood that correlate with ALS progression (Rolfes et al., 2021), and experiments of selective ablation of CD8^+^ T-cells in mice support the evidence of a dual role of CD8^+^ cells in MN degeneration (Coque et al., 2019). Surely, CD8^+^ T-cells are involved in the direct MNs killing in ALS through the MHC-I and the secretion of granzymes and perforins (Nardo et al., 2018; Coque et al., 2019), as normally required to trigger a CD8^+^-mediated response. MHC-I is expressed by MNs both physiologically and during the asymptomatic disease stage (Nardo et al., 2016), while decreasing in postmortem human spinal cord samples and in later stages of disease (Song et al., 2016). Coque et al. (2019) have observed that activated CD8^+^ T-cells infiltrate the CNS of symptomatic ALS mice models and that MN-CD8^+^ T-cell coculture of mutant SOD1-expressing CD8^+^ T cells selectively leads to MN death through MHC-I upregulation in MN, elicited by CD8^+^-derived IFN-γ stimulation. The interaction between MNs and CD8^+^ T-cells appears to depend on the presentation of self-antigens via MHC-I (Coque et al., 2019), although the specific autoantigens involved remain under investigation. Although ALS is not defined an autoimmune disease, it has been suggested that an antigen associated with ALS could trigger an adaptive autoimmune response (Rodrigues Lima-Junior et al., 2021), given the evidence of several ALS-related autoantibodies detected in ALS serum and other neurodegenerative diseases (Niebroj-Dobosz et al., 1999; Dabby et al., 2000), including anti-acetylcholine receptor (Okuyama et al., 1997), anti-L-type calcium channels (Kimura et al., 1994; Offen et al., 1998), anti-TDP-43 (Conti et al., 2021), anti-agrin (Rivner et al., 2017) and anti-low density lipoprotein receptor-related protein 4 (Tzartos et al., 2014) autoantibodies.

In this context, researchers have explored the responsiveness of CD8^+^ T cells from both ALS patients and healthy individuals to peptides derived from TDP-43 (Ramachandran et al., 2023). However, their findings indicate that TDP-43 functions as a weak autoantigen, highlighting the need for further studies to identify the relevant autoantigens involved in this process (Ramachandran et al., 2023).

In addition to direct immune cell activation, TDP-43 also induces mitochondrial dysfunction (Wang et al., 2019), leading to mitochondrial DNA leakage into the cytosol (Yu et al., 2020), and subsequent activation of the cyclic GMP-AMP synthase-stimulator of interferon genes (cGAS-STING) pathway (Yu et al., 2020), which is an immune pathway that allows for the recognition of cytosolic DNA, consequently activating host innate immunity and a type-I interferon response that exacerbates NI (Paul et al., 2021; Zhou et al., 2023). Pharmacological inhibition of cGAS-STING mitigates NI (Tan et al., 2022; Marques et al., 2024) and immune responses within the brain (Fryer et al., 2024), highlighting its potential as a therapeutic target.

Taken together, these findings point to novel therapeutic strategies to target immune responses involved in ALS pathology. Strategies targeting these immune pathways include: (i) blocking TDP-43 mitochondrial import to prevent mitochondrial DNA leakage (Wang et al., 2016); (ii) inhibiting STING signaling to diminish inflammation, which has been proven to improve motor performance (Tan et al., 2022); and (iii) enhancing autophagic clearance to promote TDP-43 degradation (Barmada et al., 2014).

Understanding the multifaceted roles of immune mechanisms and the involvement of TDP-43 dynamics is essential for developing targeted therapies that leverage their neuroprotective properties while minimizing their detrimental effects, and all these findings emphasize the need to further investigate the role of adaptive immunity in TDP-43 pathology to explore potential therapeutic strategies targeting T cell mediated NI.

## Conclusions

Recent research has increasingly highlighted that altered TDP-43, NI, IS dysregulation, and GM alterations are interconnected factors contributing to the ALS onset and progression. The mislocalization and aggregation of TDP-43 have been associated with pro-inflammatory responses both in the CNS and the periphery. Aberrant immune activation (characterized by glial cell activation, disrupted BBB integrity, and altered immune cell profiles) is observed alongside GM dysbiosis in ALS patients and animal models. Furthermore, emerging evidence indicates that GM alterations can influence NI through the modulation of systemic and local immune responses via the GBA. The disruption of microbial homeostasis appears to exacerbate TDP-43 pathology by promoting inflammatory processes and compromising cellular homeostasis mechanisms, such as autophagy. Specific bacterial metabolites, including SCFAs, have been implicated in modulating neuroimmune interactions and influencing TDP-43 aggregation dynamics. The identification of distinct microbial taxa associated with ALS progression further underscores the potential of GM composition as both a biomarker and potential therapeutic target. However, despite these advancements, the right mechanisms linking TDP-43 pathology, immune dysregulation, and GM remain unclear. It is essential to elucidate how microbial metabolites influence NI and TDP-43 aggregation. Additionally, the dysbiosis impact on gut permeability and subsequent endotoxemia warrants further investigation, as these factors may contribute to the neurodegenerative process.

Future research should focus on: (i) identifying specific microbial taxa or metabolites that modulate TDP-43 pathology and NI; (ii) deciphering the role of microbial-derived metabolites in autophagy regulation and their effects on TDP-43 clearance; (iii) investigating the impact of gut permeability alterations on systemic inflammation and CNS immune responses; (iv) developing probiotic, dietary interventions, or FMT approaches aimed at restoring microbial balance and reducing TDP-43 pathology.

A deeper understanding of the TDP-43-immunity-microbiome axis may provide crucial insights into ALS pathogenesis, leading to the development of innovative therapeutic targets to slow disease progression and improve patient outcomes. The integration of multi-omics approaches, including metagenomics, metabolomics, and transcriptomics, will be instrumental in deciphering the complex interplay between genetic, immune, and environmental factors in ALS (Blacher et al., 2019). Finally, advancing knowledge in this field may facilitate the implementation of precision medicine approaches, enabling personalized interventions tailored to individual profiles of ALS patients.

## Data Availability

*Not applicable*.
